# Interferon Alpha-2b Therapy in Chronic Hepatitis Delta

**DOI:** 10.5812/hepatmon.15729

**Published:** 2014-03-01

**Authors:** Maryam Keshvari, Seyed Moayed Alavian, Heidar Sharafi, Gharib Karimi, Mohammad Gholami Fesharaki

**Affiliations:** 1 Blood Transfusion Research Center, High Institute for Research and Education in Transfusion Medicine, Tehran, IR Iran; 2 Middle East Liver Disease (MELD) Center, Tehran, IR Iran; 3 Department of Biostatistics, School of Medical Sciences, Tarbiat Modares University, Tehran, IR Iran

**Keywords:** Hepatitis D, Interferon-alpha, Iran

## Abstract

**Background:**

Approximately 5% of hepatitis B virus (HBV) carriers are coinfected with hepatitis D virus (HDV). HBV/HDV coinfection is a major cause of cirrhosis and end stage liver disease in chronic HBsAg carriers. The only approved therapy for chronic hepatitis delta is interferon alpha (IFN α) in either pegylated or conventional forms. Although higher doses and longer durations of IFN α therapy in HBV/HDV coinfected patients are currently applied, yet treatment response is low.

**Objectives:**

We aimed to determine the efficacy of IFN α-2b therapy in patients with HBV/HDV coinfection.

**Patients and Methods:**

In this cross sectional study, 20 HBsAg carriers with positive Anti-HDVAb and RT-PCR for HDV RNA were recruited and treated for three year duration with 5 million units (MU) of IFN α-2b, three times weekly or one year with 5 MU of IFN α-2b daily. Sustained virological response (SVR) was defined as a negative qualitative HDV RT-PCR, 6 months after treatment cessation.

**Results:**

Overall, 3 (15%) subjects achieved SVR, 10 cases (50%) relapsed after treatment cessation and 7 (35%) patients did not clear HDV during the treatment.

**Conclusions:**

HDV coinfection with HBV had very low response rate to high doses and long durations of IFN α-2b therapy.

## 1. Background

Hepatitis delta virus (HDV) is a defective RNA virus that requires hepatitis B virus (HBV) in order to replicate. HBV and HDV coinfection in acute and chronic forms can lead to fulminant hepatitis, liver cirrhosis, and hepatocellular carcinoma (1, 2). About 5% of HBsAg positive patients are also infected with hepatitis delta (2). HDV infection has a worldwide distribution and is endemic in the Middle East, Mediterranean area, Amazonian region, and in many African countries (3, 4). In Iran, the prevalence rate of hepatitis D infection in HBV infected patients varies from 2.4% in blood donors to 10% in patients with chronic liver disease (5). Since hepatitis delta is a devastating form of viral hepatitis and there is no effective therapy for it, practitioners usually encounter complicated forms of chronic HDV infection (6). The only established treatment for chronic hepatitis delta (CHD) is interferon (IFN) at high doses (7). High doses of IFN (nine million units (MU) three times weekly or 5 MU daily) for at least one year led to improved outcomes as determined by biochemical and virological responses (ALT normalization and loss of HDV RNA), which correlated with improvements in liver histology (8, 9). Improved treatment response has been reported with application of pegylated interferon (PEG-IFN) in both IFN-naïve patients and previous non-responders to standard IFN therapy, thus using PEG-IFN as the first line therapy for CHD should be considered (7). In spite of higher efficacy of PEG-IFN compared to conventional IFN in CHD treatment, conventional IFN is still the selected treatment of HDV infection in developing countries mainly because of its lower costs. There is little data about CHD treatment in the literature and the current study is the first study regarding CHD treatment in Iran.

## 2. Objectives

In this study, it was aimed to assess the efficacy of IFN α-2b therapy in patients with CHD.

## 3. Patients and Methods

### 3.1. Study Population

A total of 20 HBsAg positive patients with positive anti-HDVAb and RT-PCR for HDV, who were referred to the Tehran Hepatitis Center (Tehran, Iran) and Tehran Blood Transfusion Hepatitis Clinic (Tehran, Iran) between 2008 and 2011 were enrolled to the study and treated. The CHD diagnosis was based on anti-HDVAb and HDV RNA positivity and histological findings of CHD. All patients had compensated liver disease with histological evidence of chronic hepatitis to cirrhosis. The patients were excluded if they met any of the following criteria: seropositivity for human immunodeficiency virus antibody, seropositivity for anti-HCV antibody, serious medical illness, white blood cell count less than 3,000/mm^3^, absolute neutrophil count less than 1,500/mm^3^, platelet count under 70,000/mm^3^, presence of decompensated liver disease or a history of ascites, variceal hemorrhage, hepatic encephalopathy, and hepatocellular carcinoma. Informed consent was obtained from all patients participating in the study which was approved by the Ethics Committee of the Iranian Blood Transfusion Organization. The study protocol conformed to the ethical guidelines of the 1975 declaration of Helsinki.

### 3.2. Treatment Regimen, Patient Management and Laboratory Assessments

IFN α-2b (PDferon B^®^), Pooyesh Daru, Tehran, Iran, was started with five MU three times a week for one month in order to evaluate tolerability of the treatment and patients adherence to it. In patients who tolerated the treatment, IFN α-2b dose was increased to 5 MU once daily for one year (treatment group A), and the remaining were treated with 5 MU three times weekly for 3 years (treatment group B). The treatment group B consisted of patients with severe liver disease and treatment adverse events. The patients had two physician visits in the first month and once a month in the following period of treatment. Serial hematological, biochemical, and virological studies were performed during visits.

HBeAg, anti-HBeAb, HBsAg, anti-HBsAb, anti-HDVAb were tested with the ELISA (Radim SpA, Rome, Italy) method. Plasma HBV DNA level was assessed at the beginning of treatment, at months 6, 12 and 24, at the end of treatment, and at the end of the follow-up period using COBAS Amplicore HBV Monitor Test (Roche Diagnostics). HDV RNA was detected using RT-PCR. Briefly, viral RNA was extracted from anti-HDVAb positive patients using QIAmp Viral RNA Mini kit (Qiagen, Hilden, Germany) according to the manufacturer’s instructions. The extracted RNA was subjected to reverse transcription by Revert Aid^TM^ First Strand cDNA Synthesis Kit (Fermentas, Vilnius, Lithuania). The cDNA samples were amplified using primers 8276 (5’-ACA AGG AGA GGC AGG ATC ACC GAC-3’) and 5413 (5’-GCC CAG GTG GGA CCG CGA GGA GGT-3’) for the first round of PCR and primers 5415 (5’-GAA GGA AGG CCC TCG AGA ACA AGA-3’) and 5414 (5’-GAG ATG CCA TGC CGA CCC GAA GAG-3’) for the nested round of PCR (10). All patients undergone liver biopsy and the specimens were assessed by an expert pathologist according to the modified Knodell scoring system. The biopsy results with liver fibrosis scores 0 to 4 and the ones with more than 4were categorized as mild to moderate and sever fibrosis, respectively.

### 3.3. Definition of Treatment Response

A biochemical response was defined as normal serum alanine transaminase (ALT) level (< 30 IU/L) at the end of treatment, a virological response as the absence of serum HDV RNA at the end of treatment and sustained virological response (SVR) as a negative qualitative PCR, 6 months after treatment cessation. Patients remaining HDV RNA positive at the end of treatment were considered as non-responders. Patients whom were HDV RNA negative at the end of treatment and became positive 6 months after treatment cessation were considered as relapsers.

### 3.4. Statistical Analysis

Categorical variables were expressed by frequencies and percentages. Continuous variables were expressed by mean ± standard deviation (SD). All statistical analyses were performed using SPSS software for Windows (SPSS, version 17).

## 4. Results

The study group was comprised of 20 HDV infected patients. All patients were HBsAg and anti-HBeAb positive and none were HBeAg positive. The demographic, laboratory, and histological characteristics of patients are summarized in Table 1. All patients completed the treatment course and the most observed treatment complications were anorexia, weakness, and weight loss. Twelve (60%) patients had mild to moderate liver fibrosis and 8 (40%) patients had severe liver fibrosis, from which 2 had compensated liver cirrhosis. Seven (35%) patients met the non-responder criteria, 10 (50%) relapsed, and 3 (15%) patients had SVR. Interestingly, none of the female patients were non-responder (Table 1). Also, we observed lower rate of non-response and relapse and higher rate of SVR in patients who had received longer durations of treatment (group B) than group A (Table 1). The mean ALT level was evaluated before and after treatment in non-responders, relapsers and responders. The mean ALT decreased at the end of the treatment in all response groups but it rose at the end of the follow up time (Table 1). Sixteen (80%) patients had HBV DNA less than 10^4^ copies/mL before the treatment and all patients achieved HBV DNA less than 10^4^ copies/mL at the end of the follow up time. The mean platelet count was in the normal range before the treatment and it had decreased at the end of the treatment in all response groups. HBsAg seroconversion was observed only in one female patient after completion of the treatment course.

**Table 1.** Demographic, Laboratory, and Histological Characteristics of Patients

	SVR ^a^, (n = 3)	Relapse, (n = 10)	Non-response, (n = 7)
**Gender, No. (%)**			
Male	2 (66.7)	8 (80.0)	7 (100)
Female	1 (33.3)	2 (20.0)	0 (0)
**Age, Mean ± SD, y**	43.7 ± 1.2	42.7 ± 11.6	43.1 ± 13.4
**Treatment Group, No. (%)**			
Group A	1 (33.3)	7 (70.0)	6 (85.7)
Group B	2 (66.7)	3 (30.0)	1 (14.3)
**Liver Fibrosis, No. (%)**			
Mild to moderate	2 (66.7)	5 (50.0)	5 (71.4)
Severe	1 (33.7)	5 (50.0)	2 (28.6)
**Baseline HBV DNA Level (copies/mL), No. (%)**			
< 10^4^	2 (66.7)	10 (100)	4 (57.1)
> 10^4^	1 (33.3)	0 (0)	3 (42.9)
**ALT ** ^**a**^ ** (Baseline), Mean ± SD, IU/L**	75.7 ± 66.7	79.4 ± 31.4	74.9 ± 25.0
**ALT (EOT ^a^), Mean ± SD, IU/L**	35.0 ± 17.5	53.2 ± 26.9	47.4 ± 24.1
**ALT (Follow up), Mean ± SD, IU/L**	53.3 ± 14.2	78.0 ± 53.8	57.6 ± 39.8
**AST ** ^**a**^ ** (Baseline), Mean ± SD, IU/L**	42.0 ± 7.9	77.0 ± 56.4	67.3 ± 23.4
**PLT ** ^**a**^ ** (Baseline), Mean ± SD, x10^3^/mm^3^**	138.0 ± 44.7	147.2 ± 30.7	164.7 ± 52.4

^a^ Abbreviations: ALT, alanine transaminase; AST, aspartate transaminase; EOT, end of treatment; PLT, platelet; SD, standard deviation; SVR, sustained virological response.

## 5. Discussion

HDV is an etiological agent of acute and chronic liver disease and most chronically infected patients experience severe forms of liver damage such as cirrhosis and hepatocellular carcinoma (11-13). In our study, 40% of patients had severe liver fibrosis (stage > 4). In an Italian cohort study, among 188 HDV infected patients, 82 (43%) patients had a histology of chronic hepatitis and the remaining 106 (57%) individuals had a clinical/histological diagnosis of cirrhosis (14). In another study by Farci et al. half of the patients had active cirrhosis in their liver biopsies (15). A few studies evaluated the response rate to IFN α treatment in CHD but their results are difficult to interpret because of differences in their study design and patient characterization. Additionally, most studies, which evaluated the efficacy of IFN therapy in CHD, consisted of a small number of patients (less than 25 individuals) (6, 16, 17). However, the majority of these studies showed biochemical and histological improvement but virological response had only occurred in a minority of patients (16, 18, 19). Most current antiviral agents such as nucleoside analogues inhibiting HBV replication are ineffective against HDV and combination therapy of IFN α with lamivudine or ribavirin has not shown significant advantages over monotherapy with either IFN α or PEG-IFN α (13, 20, 21). High dose IFN α is the only effective agent with limited activity against HDV (7). In our study, group B patients receiving IFN α-2b for a longer duration had higher SVR rate than group A patients who received IFN α-2b for a shorter course. In a review article, was also stated that patients may benefit more of long duration IFN treatment (7). In a study from Turkey, a two year treatment did not appear to increase sustained response rate to IFN α over one year treatments (6). Another study from Turkey showed longer duration of PEG-IFN α (2 years vs. 1 year) had no significant effect on SVR (22). Farci et al. found that patients who received higher doses of IFN α gained higher SVR rates compared to the subjects treated with lower doses of IFN α (15, 23). The main aim of the treatments is HDV RNA negativity and persistence of it during the follow up period. Among participants in this study, 12 (60%) patients lost HDV RNA at the end of treatment but this situation was stable for only 3 (15%) subjects at the end of the follow up period. In a study by Niro et al. the rate of final response (SVR) was similar (12%) to that determined in the present study (24). Also, Abbas et al. reported SVR in about 17% of IFN α treated patients (25). Interestingly, among patients achieving SVR in the current study, one female patient cleared HBV and HDV simultaneously. In a study by Lau et al. among the 6 HBV/HDV coinfected patients who completed at least 11 months of treatment with IFN α-2b, four cases lost serum HBsAg, while three of them developed anti-HBsAb (17). Some experts recommended prolonging IFN treatment duration until HBsAg loss in treatment responders and adjusting the IFN dose to patient tolerance (7, 26, 27). Recently, application of PEG-IFN α in HDV infected patients has increased virological response to about 30% (2, 24, 28) and in a study from Turkey, response rate to PEG-IFN α-2a or 2b in HDV infected patients was 47% (29). On the other hand, a recent systematic review which included randomized clinical trials of HDV treatment, indicated that the efficacy of treatment with IFN at high doses and PEG-IFN was the same (30). In another study from France, combination therapy with PEG-IFN α and a nucleoside/tide analogue seemed to be more effective than IFN α alone (31). Patients with high HBV replication may benefit from this combination therapy (32).

In the current study, 6 (30%) patients achieved normal ALT levels at the end of the treatment, in other similar studies, 20%-25% of patients were found to have normal ALT levels at the end of the treatment (4, 19). Among 6 patients with normal ALT at the end of the treatment, 5 (25%) subjects remained to have normal ALT levels during the follow up, while this rate was 26% in the study by Rosina et al.(19). Hadziyannis et al. showed that IFN α had an impact on normalization of ALT value during therapy but this effect did not last during the follow up course (33). Another finding by different studies was the suppressing effect of HDV on HBV replication (12, 34). Low HBV DNA levels before and after treatment in most of our patients confirmed this theory. However, according to the results of other studies, there are different patterns of replication in HBV/HDV co-infected patients (active HDV/inactive HBV: 70%, active HDV and HBV: 23%, inactive HDV/active HBV: 4%, both inactive: 3%) (14). The main limitation of the current study was the small sample size, which was caused by the low prevalence of HDV in Iran and exclusion of cirrhotic patients who were intolerable of IFN therapy. In conclusion, our study demonstrated that IFN α has limited effects on hepatitis D. It seems that prolonging the treatment duration has positive effects on treatment response. Further studies with larger sample sizes and newer drugs are required to evaluate the response rate.

## References

[1] Rizzetto M, Canese MG, Arico S, Crivelli O, Trepo C, Bonino F (1977). Immunofluorescence detection of new antigen-antibody system (delta/anti-delta) associated to hepatitis B virus in liver and in serum of HBsAg carriers.. Gut..

[2] Wedemeyer H, Yurdaydin C, Dalekos GN, Erhardt A, Cakaloglu Y, Degertekin H (2011). Peginterferon plus adefovir versus either drug alone for hepatitis delta.. N Engl J Med..

[3] Hughes SA, Wedemeyer H, Harrison PM (2011). Hepatitis delta virus.. Lancet..

[4] Husa P, Linhartova A, Nemecek V, Husova L (2005). Hepatitis D.. Acta Virol..

[5] Alavian S, Alavian S (2005). Hepatitis D Virus Infection; Iran, Middle East and Central Asia.. Hepat Mon..

[6] Yurdaydin C, Bozkaya H, Karaaslan H, Onder FO, Erkan OE, Yalcin K (2007). A pilot study of 2 years of interferon treatment in patients with chronic delta hepatitis.. J Viral Hepat..

[7] Farci P, Chessa L, Balestrieri C, Serra G, Lai ME (2007). Treatment of chronic hepatitis D.. J Viral Hepat..

[8] Madejon A, Cotonat T, Bartolome J, Castillo I, Carreno V (1994). Treatment of chronic hepatitis D virus infection with low and high doses of interferon-alpha 2a: utility of polymerase chain reaction in monitoring antiviral response.. Hepatology..

[9] Yurdaydin C, Idilman R, Bozkaya H, Bozdayi AM (2010). Natural history and treatment of chronic delta hepatitis.. J Viral Hepat..

[10] Smedile A, Niro MG, Rizzetto M (2004). Detection of serum HDV RNA by RT-PCR.. Methods Mol Med..

[11] Gheorghe L, Iacob S, Simionov I, Vadan R, Constantinescu I, Caruntu F (2011). Weight-based dosing regimen of peg-interferon alpha-2b for chronic hepatitis delta: a multicenter Romanian trial.. J Gastrointestin Liver Dis..

[12] Ho E, Deltenre P, Nkuize M, Delwaide J, Colle I, Michielsen P (2013). Coinfection of hepatitis B and hepatitis delta virus in Belgium: a multicenter BASL study. Prospective epidemiology and comparison with HBV mono-infection.. J Med Virol..

[13] Kaymakoglu S, Karaca C, Demir K, Poturoglu S, Danalioglu A, Badur S (2005). Alpha interferon and ribavirin combination therapy of chronic hepatitis D.. Antimicrob Agents Chemother..

[14] Niro GA, Smedile A, Ippolito AM, Ciancio A, Fontana R, Olivero A (2010). Outcome of chronic delta hepatitis in Italy: a long-term cohort study.. J Hepatol..

[15] Farci P, Mandas A, Coiana A, Lai ME, Desmet V, Van Eyken P (1994). Treatment of chronic hepatitis D with interferon alfa-2a.. N Engl J Med..

[16] Gaudin JL, Faure P, Godinot H, Gerard F, Trepo C (1995). The French experience of treatment of chronic type D hepatitis with a 12-month course of interferon alpha-2B. Results of a randomized controlled trial.. Liver..

[17] Lau JY, King R, Tibbs CJ, Catterall AP, Smith HM, Portmann BC (1993). Loss of HBsAg with interferon-alpha therapy in chronic hepatitis D virus infection.. J Med Virol..

[18] Battegay M, Simpson LH, Hoofnagle JH, Sallie R, Di Bisceglie AM (1994). Elimination of hepatitis delta virus infection after loss of hepatitis B surface antigen in patients with chronic delta hepatitis.. J Med Virol..

[19] Rosina F, Pintus C, Meschievitz C, Rizzetto M (1991). A randomized controlled trial of a 12-month course of recombinant human interferon-alpha in chronic delta (type D) hepatitis: a multicenter Italian study.. Hepatology..

[20] Gunsar F, Akarca US, Ersoz G, Kobak AC, Karasu Z, Yuce G (2005). Two-year interferon therapy with or without ribavirin in chronic delta hepatitis.. Antivir Ther..

[21] Yurdaydin C, Bozkaya H, Onder FO, Senturk H, Karaaslan H, Akdogan M (2008). Treatment of chronic delta hepatitis with lamivudine vs lamivudine + interferon vs interferon.. J Viral Hepat..

[22] Gulsun S, Tekin R, Bozkurt F (2011). Treatment of chronic delta hepatitis: a nine-year retrospective analysis.. Hepat Mon..

[23] Farci P, Roskams T, Chessa L, Peddis G, Mazzoleni AP, Scioscia R (2004). Long-term benefit of interferon alpha therapy of chronic hepatitis D: regression of advanced hepatic fibrosis.. Gastroenterology..

[24] Niro GA, Ciancio A, Gaeta GB, Smedile A, Marrone A, Olivero A (2006). Pegylated interferon alpha-2b as monotherapy or in combination with ribavirin in chronic hepatitis delta.. Hepatology..

[25] Abbas Z, Khan MA, Salih M, Jafri W (2011). Interferon alpha for chronic hepatitis D.. Cochrane Database Syst Rev..

[26] Brichler S, Setshedi M, Renou C (2013). Resolution of chronic hepatitis delta infection after five years of peginterferon-adefovir: lessons from a case report.. Clin Res Hepatol Gastroenterol..

[27] Lau DT, Kleiner DE, Park Y, Di Bisceglie AM, Hoofnagle JH (1999). Resolution of chronic delta hepatitis after 12 years of interferon alfa therapy.. Gastroenterology..

[28] Alavian SM, Tabatabaei SV, Behnava B, Rizzetto M (2012). Standard and pegylated interferon therapy of HDV infection: A systematic review and meta- analysis.. J Res Med Sci..

[29] Karaca C, Soyer OM, Baran B, Ormeci AC, Gokturk S, Aydin E (2013). Efficacy of pegylated interferon-alpha treatment for 24 months in chronic delta hepatitis and predictors of response.. Antivir Ther..

[30] Lamers MH, Kirgiz OO, Heidrich B, Wedemeyer H, Drenth JP (2012). Interferon-alpha for patients with chronic hepatitis delta: a systematic review of randomized clinical trials.. Antivir Ther..

[31] Mansour W, Ducancelle A, Le Gal F, Le Guillou-Guillemette H, Abgueguen P, Pivert A (2010). Resolution of chronic hepatitis Delta after 1 year of combined therapy with pegylated interferon, tenofovir and emtricitabine.. J Clin Virol..

[32] Dastgerdi ES, Herbers U, Tacke F (2012). Molecular and clinical aspects of hepatitis D virus infections.. World J Virol..

[33] Hadziyannis SJ (1991). Use of alpha-interferon in the treatment of chronic delta hepatitis.. J Hepatol..

[34] Gish RG, Yi DH, Kane S, Clark M, Mangahas M, Baqai S (2013). Coinfection with hepatitis B and D: epidemiology, prevalence and disease in patients in Northern California.. J Gastroenterol Hepatol..

